# SiMeEx, a simplified method for metabolite extraction of adherent mammalian cells

**DOI:** 10.3389/fmolb.2022.1084060

**Published:** 2022-12-21

**Authors:** Antonia Henne, Anna Vigh, Andre Märtens, Yannic Nonnenmacher, Melanie Ohm, Shirin Hosseini, Tushar H. More, Mario A. Lauterbach, Hendrikus Garritsen, Martin Korte, Wei He, Karsten Hiller

**Affiliations:** ^1^ Department of Bioinformatics and Biochemistry, Braunschweig Integrated Centre of Systems Biology, Technische Universität Braunschweig, Braunschweig, Germany; ^2^ Department of Cellular Neurobiology, Zoological Institute, Technische Universität Braunschweig, Braunschweig, Germany; ^3^ Institute of Transfusion Medicine, Klinikum Braunschweig, Braunschweig, Germany; ^4^ Fraunhofer Institute for Surface Engineering and Thin Films IST, Braunschweig, Germany; ^5^ Neuroinflammation and Neurodegeneration Research Group, Helmholtz Centre for Infection Research, Braunschweig, Germany

**Keywords:** mammalian cells, GC-MS, metabolite extraction, metabolomics, stable isotope labeling

## Abstract

A reliable method for metabolite extraction is central to mass spectrometry-based metabolomics. However, existing methods are lengthy, mostly due to the step of scraping cells from cell culture vessels, which restricts metabolomics in broader application such as lower cell numbers and high-throughput studies. Here, we present a simplified metabolite extraction (SiMeEx) method, to efficiently and quickly extract metabolites from adherent mammalian cells. Our method excludes the cell scraping step and therefore allows for a more efficient extraction of polar metabolites in less than 30 min per 12-well plate. We demonstrate that SiMeEx achieves the same metabolite recovery as using a standard method containing a scraping step, in various immortalized and primary cells. Omitting cell scraping does not compromise the performance of non-targeted and targeted GC-MS analysis, but enables metabolome analysis of cell culture on smaller well sizes down to 96-well plates. Therefore, SiMeEx demonstrates advantages not only on time and resources, but also on the applicability in high-throughput studies.

## 1 Introduction

Metabolomics has emerged as a new branch of the *-omics* science and aims to identify the entirety of metabolites, termed as metabolome, in a given sample from cells, tissues or organs (Vailati-Riboni et al., 2017). The metabolome includes endogenous and exogenous small molecules, consisting among others of sugars, amino acids, lipids and amines, which are all essential for the function of biological systems (Clish, 2015; Allison, 2017). To identify the plethora of different metabolites, specific analytical tools are required. Commonly used spectrometric techniques are nuclear magnetic resonance (NMR) and mass spectrometry (MS). Each method has its own pros and cons, while MS prevails in sensitivity and selectivity (Emwas, 2015). In most cases it is coupled with gas- or liquid-chromatography (GC-MS; LC-MS) to achieve a conspicuous separation of metabolites before ionization. Particularly, GC-MS established itself as a robust method for the analysis of metabolites of central carbon metabolism and thereby is routinely employed in laboratories all over the world (Zhang et al., 2012).

A precise and yet fast sample processing prior to GC-MS measurement is key to reliable results, irrespective of samples from prokaryotes, plants or animal cells (Roessner et al., 2000; Buchholz et al., 2002; Villas-Bôas et al., 2005; Alseekh et al., 2021). For adherent mammalian cells in culture the normal procedure involves their detachment from the surface. This can be achieved by either a treatment with trypsin/ethylenediaminetetraacetic acid (EDTA) or by simply scraping the cells of the plate (Hutschenreuther et al., 2012). Since detachment *via* trypsin or EDTA can lead to an alteration of cell metabolism and trypsinization of cells can cause leakage of the cell membrane, most commonly the cells are first quenched with an organic solvent, followed by cell scraping (García-Cañaveras et al., 2016; Luo et al., 2020). For reproducibility the quenching step needs to be performed as fast as possible to stop all enzymatic activities and metabolite conversions and to prevent the degradation of existing cellular metabolites (Dettmer, 2011; Alseekh et al., 2021). For such aims, several attempts were made to improve metabolite extraction efficiency (Teng et al., 2009; Dettmer, 2011; Sapcariu et al., 2014; Madji Hounoum et al., 2015; Ser et al., 2015; García-Cañaveras et al., 2016). However, all these efforts share a common cell-scraping step, which limits further optimization of the extraction efficiency. In this current study, we present a new and shorter method, termed as simplified metabolite extraction (SiMeEx), to extract metabolites from adherent mammalian cells. Our new method excludes the tedious and error-prone scraping step and therefore allows for a more efficient and reproducible extraction of polar metabolites in less than 30 min per 12-well plate. We demonstrate that SiMeEx achieves the same metabolite recovery in various immortalized and primary cells as other methods that involve a scraping step. Furthermore, we show that omitting cell scraping does not interfere with the performance of non-targeted and targeted GC-MS analysis. Finally, we tested SiMeEx in smaller well sizes down to 96-well plates and demonstrate its applicability in high-throughput studies.

## 2 Materials and methods

### 2.1 Reagent and chemicals

If not stated otherwise, all reagents were purchased from Sigma-Aldrich (Steinheim, Germany). For reagents used for metabolite extraction, HPLC grade was used.

### 2.2 Cell culture

Mouse macrophage RAW 264.7 cells were cultured in RPMI 1640 medium (Gibco™, 21875034, Thermo Fisher Scientific™, Waltham, United States). Human pulmonary carcinoma epithelial A549 cells, human colon epithelial HT-29 cells and embryonic mouse fibroblast NIH3T3 cells were cultured in DMEM medium (Gibco™, 41965039, Thermo Fisher Scientific™, Waltham, United States). Human monocyte-derived macrophages (hMDMs) were isolated from buffy coats *via* density gradient centrifugation with Biocoll (Bio & Sell GmbH, Feucht, Germany) followed by selection with anti-CD14 microbeads (Miltenyi Biotec, Bergisch Gladbach, Germany) and differentiation with 50 U/ml human M-CSF (ImmunoTools GmbH, Friesoythe, Germany) in RPMI 1640 medium for 6–7 days. Buffy coats were obtained from healthy donors according to protocols accepted by the Landesärztekammer Niedersachsen (ethics votes Bo/64/2021). Mouse bone marrow derived macrophages (BMDMs) were isolated from hind leg bones of C57Bl/6JolaHsd mice (Harlan/Envigo) and differentiated in DMEM medium with 25 ng/ml mouse M-CSF (Miltenyi Biotec, Bergisch Gladbach, Germany) added for 6–7 days. The mice were bred and kept at the animal facility of the TU Braunschweig in accordance with animal guidelines under standard housing conditions in a 12-h light:dark cycle at 22°C with food and water available *ad libitum*. All procedures concerning animals were approved by the animal welfare representative of the TU Braunschweig and the LAVES (Oldenburg, Germany, Az. §4 (02.05) TSchB TU BS), and ethical review and approval were not required for this study in accordance with §4 (3) of the German Animal Protection Act. All media were supplemented with 10% heat-inactivated FBS (Gibco, 10082147, Thermo Fisher Scientific™, Waltham, United States) and 1% (v/v) penicillin/streptomycine (Gibco™, 15140122, Thermo Fisher Scientific™, Waltham, United States). Cells were cultured in a humidified incubator at 37°C and 5% CO_2_.

### 2.3 Analysis of metabolites and stable isotope tracing

For comparison of the two different extraction methods, all cell types, except for NIH3T3 cells, were seeded in 12-well plates with 3 × 10^5^ (RAW 264.7), 2 × 10^5^ (A549, HT-29) or 1 × 10^6^ (hMDM, BMDM) cells per well. NIH3T3 cells were seeded in 6-well plates with 2.5 × 10^5^ cells per well. Approximately 24 h after seeding metabolite extraction (see below) was performed. For measurement of the remaining enzymatic activity after cell quenching 1 mM [U-^13^C_3_]-sodium pyruvate or unlabeled sodium pyruvate was added to ddH_2_O with 1 *μ*g/ml pentanedioic-d_6_ acid (as internal standard) and metabolite extraction was performed. For stable isotope tracing RAW 264.7 cells were seeded in 12-well plates with 2 × 10^5^ cells per well. After incubation for 18 h, medium was changed to SILAC RPMI 1640 Medium (Gibco™, A2494401, Thermo Fisher Scientific™, Waltham, United States) supplemented with 1.15 mM l-Arginine, 0.22 mM l-Lysine hydrochloride, 2 mM l-Glutamine and 11,1 mM [U-^13^C_6_]-glucose (Cambridge Isotope Laboratories, Tewksbury, United States). The FBS was exchanged to its dialyzed variant. The cells were incubated for 18 h before extraction. For 48-well or 96-well plates, 10 × 10^4^ or 5 × 10^4^ cells/well were seeded, respectively.

### 2.4 LIVE–DEAD viability assay

To assess the efficiency to terminate cell activity by applying methanol (MeOH) alone followed by ddH_2_O, we measured the cell viability in RAW 264.7 macrophages by using the LIVE/DEAD Cytotoxicity/Viability Assay Kit (Invitrogen™) according to the manufacturer’s instructions as recently described (Nonnenmacher et al., 2017). Briefly, cells were washed with 0.9% NaCl, followed by addition of 0.9% NaCl (Ctrl), ddH_2_O, MeOH or MeOH + ddH_2_O. After removal of these reagents the cells were incubated with 2 *μM* calcein-AM and 4 *μM* ethidium-homodimer 1 in 0.9% NaCl for 30 min at 37°C. Images were taken using a Zeiss Axiovert 135 TV Inverted Fluorescence Phase Contrast Microscope equipped with a ×40 objective and a Nikon DS-Fi3 camera coupled/linked to a Nis-Elements Imaging.

### 2.5 Metabolite extraction

**FIGURE 1:**
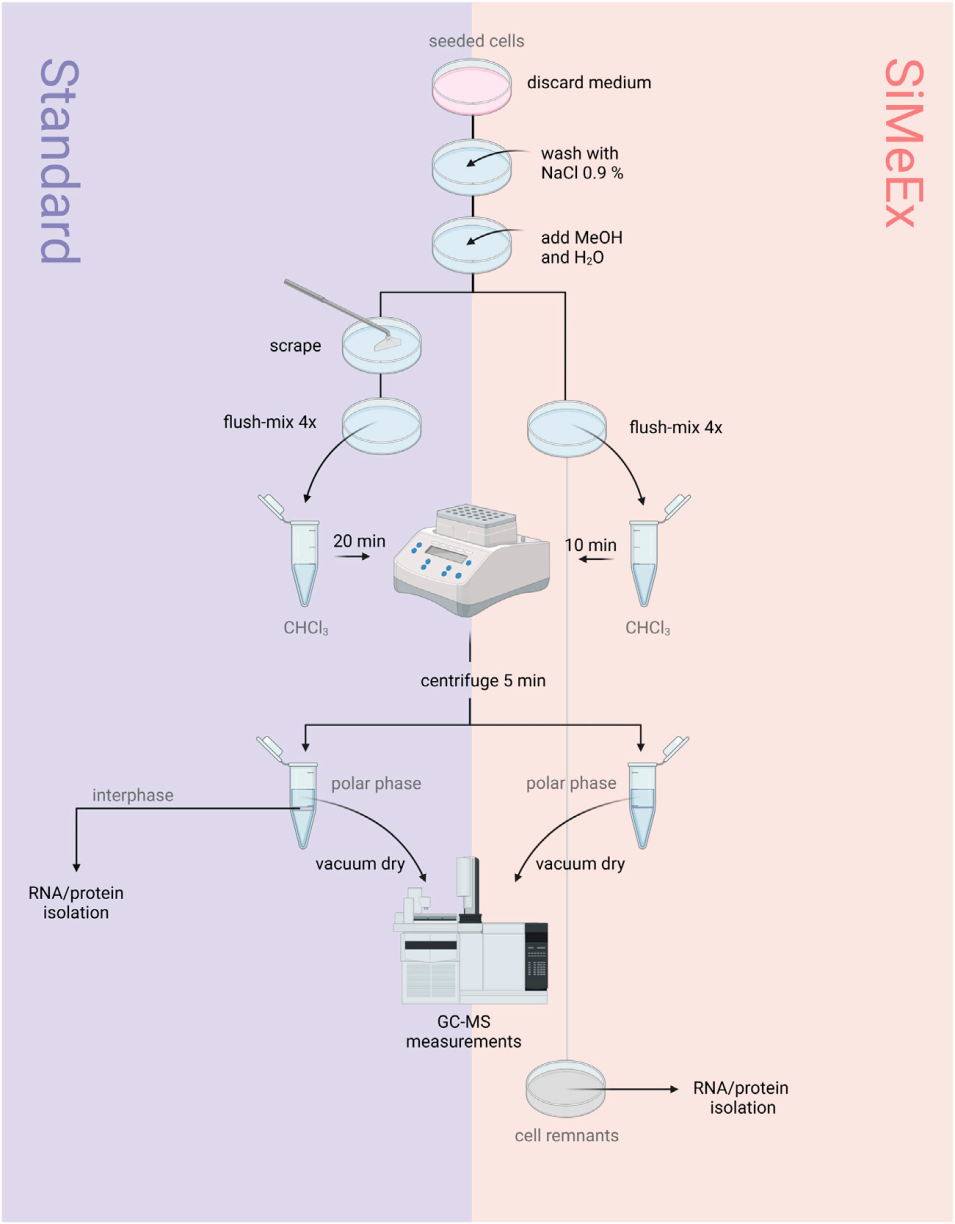
‘Standard’ vs SiMeEx. Schematic diagram of the workflows for the ‘standard’ (left) and SiMeEx (right) metabolite extraction.

The *‘standard’* procedure of metabolite extraction was performed as previously described (Sapcariu et al., 2014). The SiMeEx method compared to the *‘standard’* extraction is schematically shown in Figure 1. Briefly, the cells were washed with 0.9% NaCl solution, prior to quenching cells with equal amounts of ice-cold MeOH and ddH_2_O containing 1 *μg*/ml pentanedioic-d6 acid (as an internal standard), while the plates were maintained on an ice-cold metal plate. For the *‘standard’* method the cells were thoroughly scraped and flush-mixed four times before transferring the extraction fluid into a microtube pre-filled with cold chloroform (CHCl_3_) while the SiMeEx method excluded scraping and therefore flush-mixing was performed immediately after adding MeOH and ddH_2_O. Afterwards, microtubes were vortexed at 1400 rpm for 20 min (*‘Standard’*) or 10 min (SiMeEx), followed by centrifugation at 17,000 g for 5 min, both at 4°C. For both methods the resulting upper phase (polar phase) was transferred into a GC-compatible glass vial with a micro insert and lyophilized using a CentriVap (Labconco Corporation, Kansas City, United States). The dried samples were capped and stored at 4°C until measurement. The interphase was used for RNA and protein extraction. For metabolite extraction from 48-well and 96-well plates, adding CHCl_3_ was omitted and vortexing is shortened to 5 min at 1400 rpm. This resulted in a single polar phase and a pellet of protein and RNA. Depending on the well size used for cell culture, the volumes of added MeOH, ddH_2_O and CHCl_3_ (if used) were as follows: 300 *μL* (6-well plate), 250 *μL* (12-well plate), 160 *μL* (48-well plate) or 80 *μL* (96-well plate).

### 2.6 Protein extraction

For protein quantification, M-PER lysis buffer (Thermo Fisher Scientific™, Waltham, United States) was added to cell remnants on the well or the resulting interphase after phase separation. Protein quantification was performed by BCA Assay using the BCA Protein Assay Kit (Pierce™, Thermo Fisher Scientific™, Waltham, United States). Absorbance was measured at 562 nm and protein concentration was calculated by the standard curve.

### 2.7 RNA isolation

Isolation of total RNA from the cell remnants on the well or to the resulting interphase after phase separation was performed with the NucleoSpin^®^ RNA isolation kit (Macherey-Nagel, Düren, Germany). RNA was quantified using a NanoQuant Plate™ and the Spark^®^ multimode microplate reader (Tecan Trading AG, Männedorf, Switzerland).

### 2.8 GC-MS measurement

For comparison of metabolite levels and efficiency of the extraction method GC-MS measurement was performed. Therefore, the dried samples were first derivatized with equal amounts of methoxyamine hydrochloride (20 mg/ml in pyridin) and N-methyl-N-(tert-butyldimethylsilyl)trifluoroacetamide (MTBSTFA). For measurement of polar metabolites, 1 *μL* sample was injected in a SSL injector at 270°C in splitless mode. A 7890A GC System from Agilent coupled to a 5975C inert XL MSD was used for GC-MS analysis, equipped with a 30 m ZB-35 column from Zebron. Helium was used as a carrier gas with a flow rate of 1 ml/min. The initial oven temperature of 100°C was held for 2 min. Subsequent the temperature was ramped up to 300°C with 10°C/min and held for 4 min. Metabolites were either detected in selected ion monitoring (SIM) or full scan mode. The MSD was operating under electron ionization at 70 eV. Data processing of the chromatograms for obtaining relative metabolite levels and analysis of mass isotopomer distributions was performed using the Metabolite Detector software (Hiller et al., 2009). Data was normalized to the internal standard.

### 2.9 Statistical analysis

If not stated otherwise, all data were presented as mean ± SEM calculated from three independent experiments with minimum n = 3 technical replicates. Comparison of groups was performed by unpaired Students *t*-test. P values with *p* < 0.05 were considered statistical significant. Density plot and PCA calculation was performed with the package ‘stats’ for R. Other figures were generated by Matplolib for python and ggplot2 for R.

## 3 Results

### 3.1 Quenching with methanol is sufficient to disrupt cell membranes and terminate metabolic activity

**FIGURE 2:**
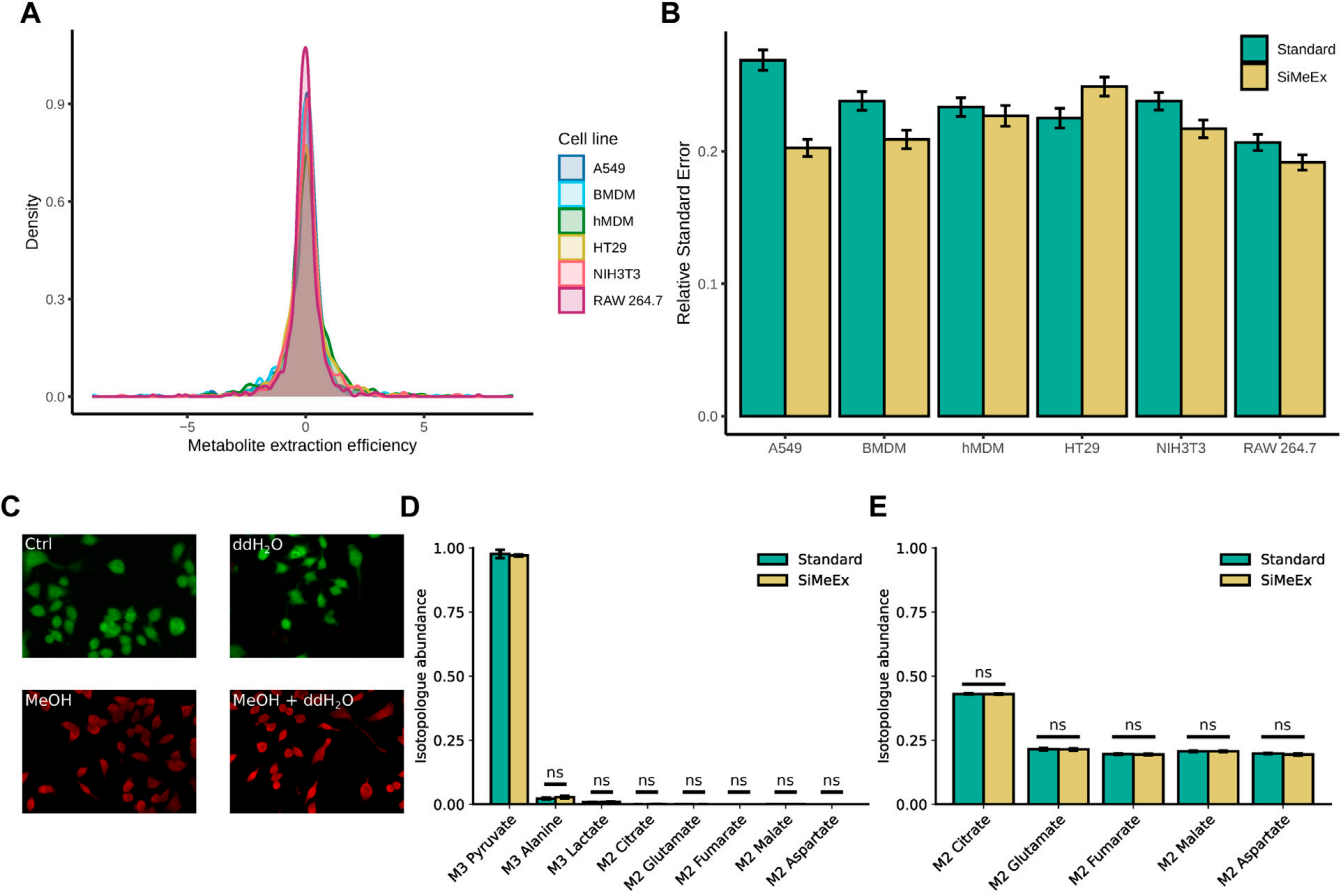
SiMeEx is sufficient to stop metabolic activities and disrupt cellular and mitochondrial membranes. **(A)**. Density plot of the metabolite extraction efficiency based on non-targeted GC-MS measurement. The metabolite extraction efficiency is defined as the log2-fold change of SiMeEx and the ‘standard’ method for the mean signals of each detected metabolite and biological replicate. **(B)**. Comparison of relative standard errors during the extraction with SiMeEx and the ‘*standard*’ method. Means and SEM are shown for each cell line, calculated based on the non-targeted measurement. **(C)**. Live/dead staining with calcein-AM (green, for esterase activity) and ethidium homodimer-1 (red, for DNA binding) of RAW 264.7 cells treated with 0.9% NaCl (Ctrl), ddH_2_O, MeOH or MeOH + ddH_2_O. **(D)**. Abundance of isotopologues of central carbon metabolites in disrupted RAW 264.7 cells incubated with [U-^13^C_3_]-sodium pyruvate. Data is normalized to isotopologue abundance from unlabeled pyruvate control samples. **(E)**. Abundance of isotopologues of mitochondrial metabolites in RAW 264.7 cells incubated with [U-^13^C_6_]-glucose, followed by *‘standard’* or SiMeEx extraction. **(B,D,E)**, data are presented as mean ± SEM pooled from three independent experiments with n = 6–12 (RAW264.7 cells) or 3 (all other cells) technical replicates each.

An efficient release of cellular metabolites is of outmost importance for all reliable extraction methods. To evaluate the performance of SiMeEx, we compared its extraction efficiency and accuracy with the method described by Sapcariu et al. (Sapcariu et al., 2014) which is widely applied and very similar to other commonly used methods (Metallo et al., 2012; Fendt et al., 2013; Christen et al., 2016; Muthusamy et al., 2020). For simplicity we denominate this method as *‘standard’* in the following. We first aimed to verify the extraction efficiency of SiMeEx in terms of metabolite recovery. For this purpose, we applied both extraction methods to extract intracellular metabolites from four different cancer cell lines (RAW 264.7, A549, HT-29 and NIH3T3 cells) and two different types of primary cells (hMDM and BMDM). These cell lines/types cover a broad range of membrane compositions that could impact the extraction efficiency of SiMeEx. After derivatization and GC-MS measurement, we calculated the ratio of the recorded signal intensities for all metabolites and cell lines (Harayama and Riezman, 2018). We found, that the metabolite extraction efficiency between SiMeEx and the *‘standard’* method was consistently similar, and independent of the employed cell type, both for non-targeted (Figure 2A) and targeted (Supplementary Figure S1A) GC-MS analysis. These findings were further supported by a principal component analysis (PCA), which shows that both methods cluster together (Supplementary Figure S2 (non-targeted), Supplementary Figure S3 (targeted)). In addition, we determined the relative standard error based on three technical replicates (except for six to twelve replicates for RAW264.7 cells) for both methods to estimate the reproducibility of the extraction methods. Likewise, SiMeEx performed similar or even better than the *‘standard’* method (Figure 2B, Supplementary Figure S1B). After confirming that SiMeEx induced membrane disruption and metabolite release, we performed further validation experiments with RAW 264.7 cells.

Next, we were interested to investigate the impact of MeOH for membrane disruption and treated RAW 264.7 cells with either 0.9% NaCl, distilled water or a mixture of distilled water and methanol (MeOH), followed by a live/dead cell staining. Indeed, the addition of MeOH to the extraction fluid is essential to disrupt cell membranes and to quench cellular metabolism (Figure 2C). To directly test if enzymatic metabolite conversion is completely halted by the employed mixture of MeOH/water, we added a [U-^13^C_3_]-pyruvate tracer to the extraction fluid. In case of any residual enzymatic activity during the extraction procedure, there would be isotope incorporation in downstream metabolites such as lactate, alanine or citrate. In fact, for both methods we detected 1% labeled lactate and 3% labeled alanine, indicating a very low level of residual activity of lactate dehydrogenase (LDH) and alanine transaminase, whereas all other metabolites had less than 1% labeling (Figure 2D).

Upon demonstrating that MeOH is sufficient for a general cell disruption without any additional cell scraping, we next asked if membranes of subcellular organelles such as mitochondria are efficiently disrupted and hence a complete release of their metabolic content. To evaluate if SiMeEx and the *‘standard’* method differ in their efficiency to liberate subcellular metabolite pools, we employed a [U-^13^C_6_]-glucose tracer to label central carbon metabolism. In case of an incomplete organelle disruption, we would expect differences in enrichment patterns of TCA cycle intermediates, because of the subcellular compartmentalization of the metabolite pools. As expected, we did not observe any difference in isotopologue abundances for the analyzed metabolites between SiMeEx and the *‘standard*’ method, showcasing a complete disruption of the cells (Figure 2E).

### 3.2 Co-extraction of nucleic acids and proteins

**FIGURE 3:**
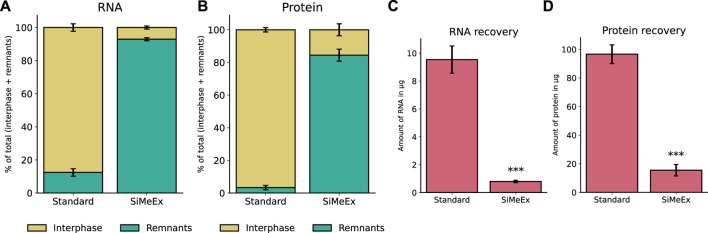
Co-extraction of nucleic acids and proteins. **(A,B)**. Distribution of recovered RNA **(A)** and protein **(B)** between interphase of extraction fluids (Interphase) and cellular remnants after extraction (Remnants), using the *‘standard’* and SiMeEx methods, and the data present the percentage distribution of Interphase and Remnants. **(C,D)**. Yield of RNA **(C)** and protein **(D)** recovered from interphase of extraction fluids, using *‘standard’* or SiMeEx methods. All data are presented as mean ± SEM calculated from three independent experiments with n = 3 technical replicates. Significances were calculated by unpaired student’s *t*-test with *p* < 0.001: ***.

A key advantage of liquid-liquid extraction methods for metabolomics is the co-extraction of nucleic acids and proteins along with polar and non-polar metabolites (Sapcariu et al., 2014; Coman et al., 2016). During phase separation, these bio-polymers are located in the interphase between the polar and non-polar solvent. Since SiMeEx excludes the scraping of adherent cell material, we assumed a lower efficiency for the co-extraction of the mentioned molecule types. We applied both methods to extract biomolecules from RAW 264.7 cells and isolated RNA and proteins from the interphase. In addition, we isolated proteins and RNA from the remainings in the well after extraction. We found that in case of SiMeEx only 7% of the RNA and 16% of the proteins were co-extracted and recovered from the interphase instead of 88% of RNA and 97% of protein in case of the *‘standard’* method (Figures 3A,B). By comparing the total amount of RNA and protein in the interphase this becomes even clearer. Only 0.7 *μg* of RNA can be recovered with SiMeEx compared with 8 *μg* with the ‘*standard*’ method (Figure 3C). Similarly, 16 *μg* protein was recovered from the interphase using SiMeEx, compared with 97 *μg* using the ‘*standard*’ method (Figure 3D). This concludes that SiMeEx is not the method of choice if a co-extraction of bio-polymers is required.

### 3.3 Potential for high-throughput applications

**FIGURE 4:**
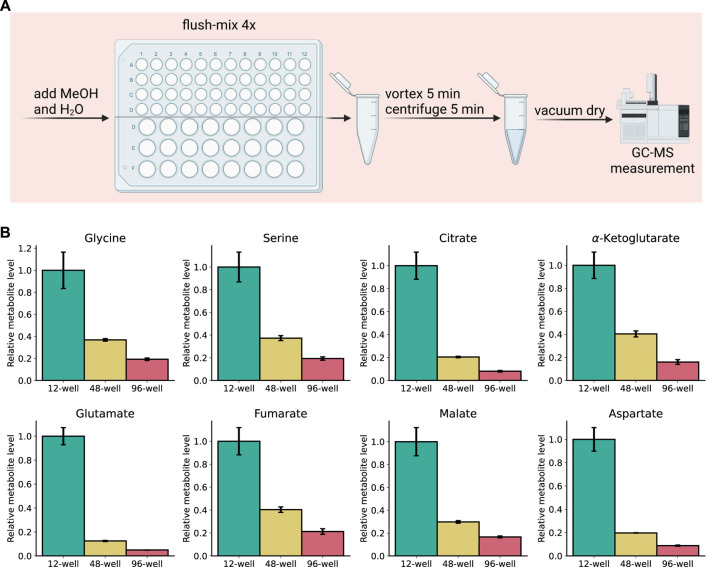
High-throughput applicability of SiMeEx. **(A)**. Schematic workflow for SiMeEx extraction for 48- or 96-well plate formats. **(B)**. Levels of representative metabolites extracted from RAW 264.7 cells cultured on different formats of multi-well plates. All data are normalized to metabolite levels of 12-well plate and presented as mean ± SEM calculated from three independent experiments with n = 3 technical replicates.

The SiMeEx method to extract metabolites of adherent cells exhibits an equal efficacy to the *‘standard’* method. Excluding the scraping step not only comes with a reduction of extraction time, but also a compatibility with smaller well sizes—both of which raise the potential for high-throughput applications. Therefore, we next aimed to test SiMeEx for RAW264.7 cells cultivated in the format of 48- and 96-well plates. To further reduce the extraction time, we omitted the addition of chloroform and subsequent phase separation, both of which are dispensable for extraction of polar metabolites. Concomitantly, the vortexing step was decreased to 5 min. A brief scheme of the extraction workflow is shown in (Figure 4A). We performed targeted metabolomics and compared levels of representative metabolites extracted from different formats of multi-well plates. As expected, samples from smaller wells exhibited lower metabolite levels acquired; specifically, metabolite levels (signal intensity) from 48-well and 96-well plates were 20–40% and 5–20% respectively, of those from a 12-well plate (Figure 4B). Despite the lower metabolite levels, the SiMeEx method manifests its applicability in high-throughput measurement of intracellular polar metabolites.

## 4 Discussion

A reliable and time-saving method for metabolite extraction is essential for an effective metabolomics analysis. Here we introduce SiMeEx, a simplified extraction method, which skips cell scraping and therefore shortens extraction time by almost half and even enables compatibility with multi-well plates of smaller sizes, while maintaining a similar performance in GC-MS measurement compared to the *‘standard’* method of metabolite extraction. In addition, skipping the scraping step also reduces plastic usage in the laboratory, which saves cost and contributes to environmental sustainability.

SiMeEx was tested with six different cell types which originated from different tissues, as well as a mix of cell lines and primary cells. We showed that the membrane composition of different cell types does not interfere with the metabolite extraction efficiency of SiMeEX, indicated by an unchanged metabolite recovery. Since cell scraping assumes some experience to ensure a satisfactory coverage of the well surface, inconsistency between experimenters is inevitable, causing undesirable variations between intra- and inter-studies. Our SiMeEx method avoids this step and hereby provides more reliability in data interpretation. Yet, before using this method for other cell types we recommend to test its extraction efficiency beforehand. Furthermore, we demonstrated with a live/dead viability assay and [U-^13^C_3_]-pyruvate tracing, that SiMeEx disrupts not only the cellular membrane, but also stops cellular activities, guaranteeing a timely quenching of cellular components. In addition, a [U-^13^C_6_]-glucose tracing experiment exhibited very similar results in the isotopic enrichment patterns of TCA cycle metabolites compared to the *‘standard’* method, corroborating the desired efficacy to extract mitochondrial metabolites as well with our simplified method.

Co-extraction of nucleic acids and proteins from the interphase between extraction solvents is a common practice of the *‘standard’* method. As we omit the cell detachment step, it is not surprising that much lower fractions of RNA and protein are recovered from the interphase. However, as most of the cellular remnants are maintained on the cell culture vessels, standard methods of extracting DNA/RNA/protein can be employed whenever required to extract these bio-macromolecules from the plate (Figures 3A,B). Therefore, our SiMeEx method is also compatible to a combined analysis of metabolites and genes/proteins, which is especially vital in the era of multi-omics.

Metabolite extraction without scraping expands the well sizes which can be used. A 24-well plate format is usually the smallest size allowing for cell scraping, while with the SiMeEx method, we extend to 48-well and 96-well formats, with satisfying metabolomics results despite expectedly lower signal intensity. Hence, this enables applicability of metabolomics in high-throughput studies where small well sizes are a normality. The option to omit chloroform and phase separation further substantiates this, whenever only polar metabolites are of interest. Additionally, absence of chloroform reduces the risk of exposure to toxic solvents in a lab, alongside a better environmental friendliness. Due to the relatively lower signal intensity, we would nevertheless recommend preliminary experiments to determine a sufficient signal intensity for target metabolites.

Taken together, SiMeEx is a simplied method to extract metabolites from cultured adherent cells. While retaining extraction efficacy and without interference on GC-MS performance, it significantly reduces extraction time and allows for cell culture on smaller well sizes. Therefore, it exhibits prominent advantages not only on time and resources, but also on the applicability in high-throughput studies.

## Data Availability

The raw data supporting the conclusion of this article will be made available by the authors, without undue reservation.
