# Supplementary material for: SiMeEx, a simplified method for metabolite extraction of adherent mammalian cells
Source: Front Mol Biosci. 2022 Dec 21;9:1084060. doi: 10.3389/fmolb.2022.1084060 (PMC9812552; doi:10.3389/fmolb.2022.1084060)
Supplement: Supplementary file 1 [file DataSheet1.pdf]

Supplementary Material

1 SUPPLEMENTARY FIGURES

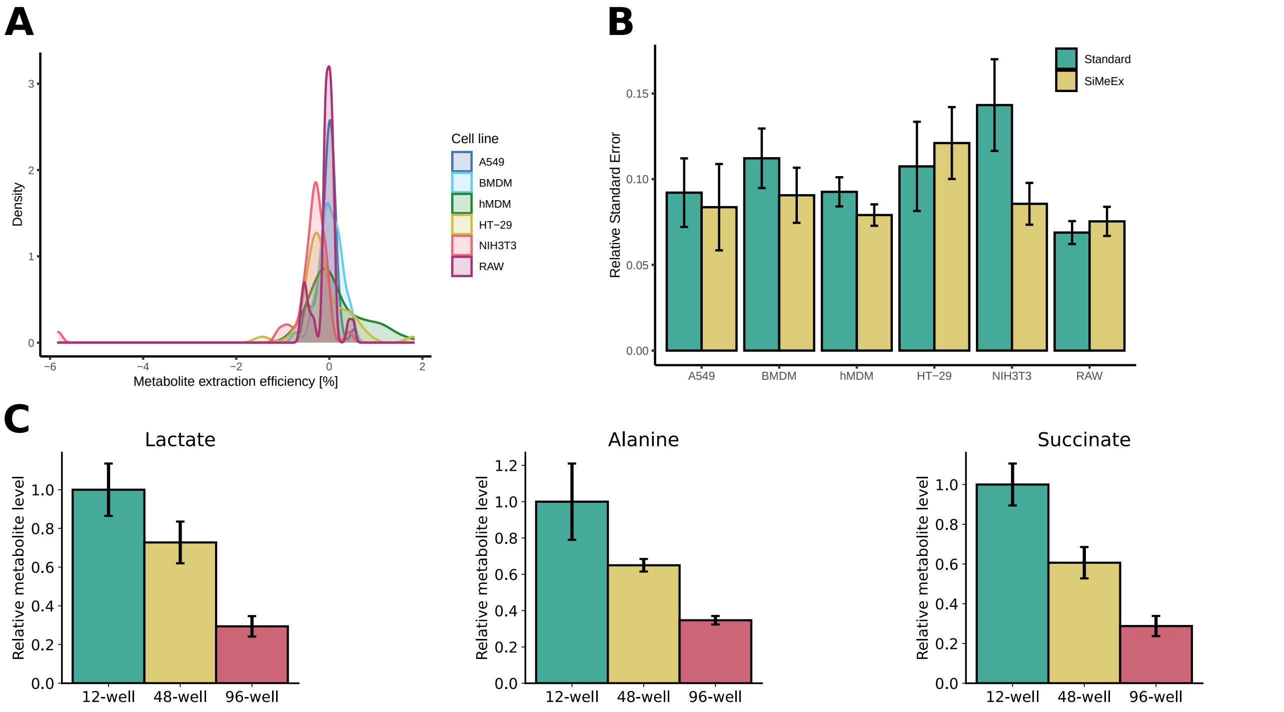

Figure S1. Efficiency of SiMeEx extraction. A. Density plot of the metabolite extraction efficiency based on targeted GC-MS measurement. The metabolite extraction efficiency is defined as the log2-fold change of the SiMeEx and *'standard'* for the mean signals of each detected metabolite and biological replicate. **B**.Comparison of relative standard errors during the extraction with SiMeEx and *'standard'*. Means and SEM are shown for each cell line, calculated based on the targeted measurement. **C**. Comparison of relative metabolite levels for metabolites after extraction with SiMeEx high throughput. **B,C**, data are presented as mean ± SEM pooled from 3 independent experiments with n=3-6 technical replicates each.

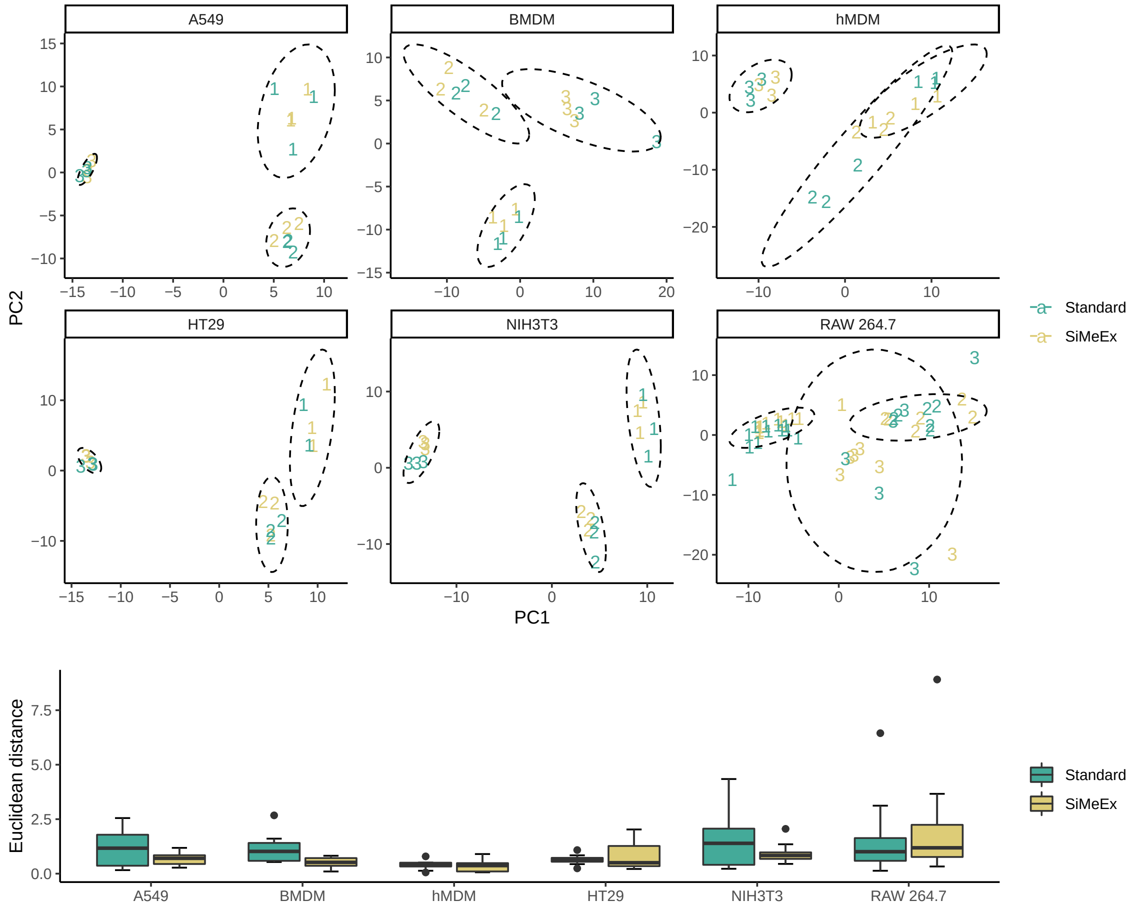

Figure S2. PCA for different cells, untargeted analysis. PCA analysis of the extraction methods. Data of an untargeted measurement with 274 metabolites as features is used, normalized by internal standard and sum of metabolite signals. Upper plots show scatter plots for cell lines. The colors depict the extraction method and the numbers indicate the biological replicates (12 for RAW264.7 cells and 3 for all other cell lines and primary cells. Ellipse are shown with dashed lines, where the biological replicate is clustered. The reproducibility of both methods is quantified, using the euclidean distance to the group centroid for each biological experiment.

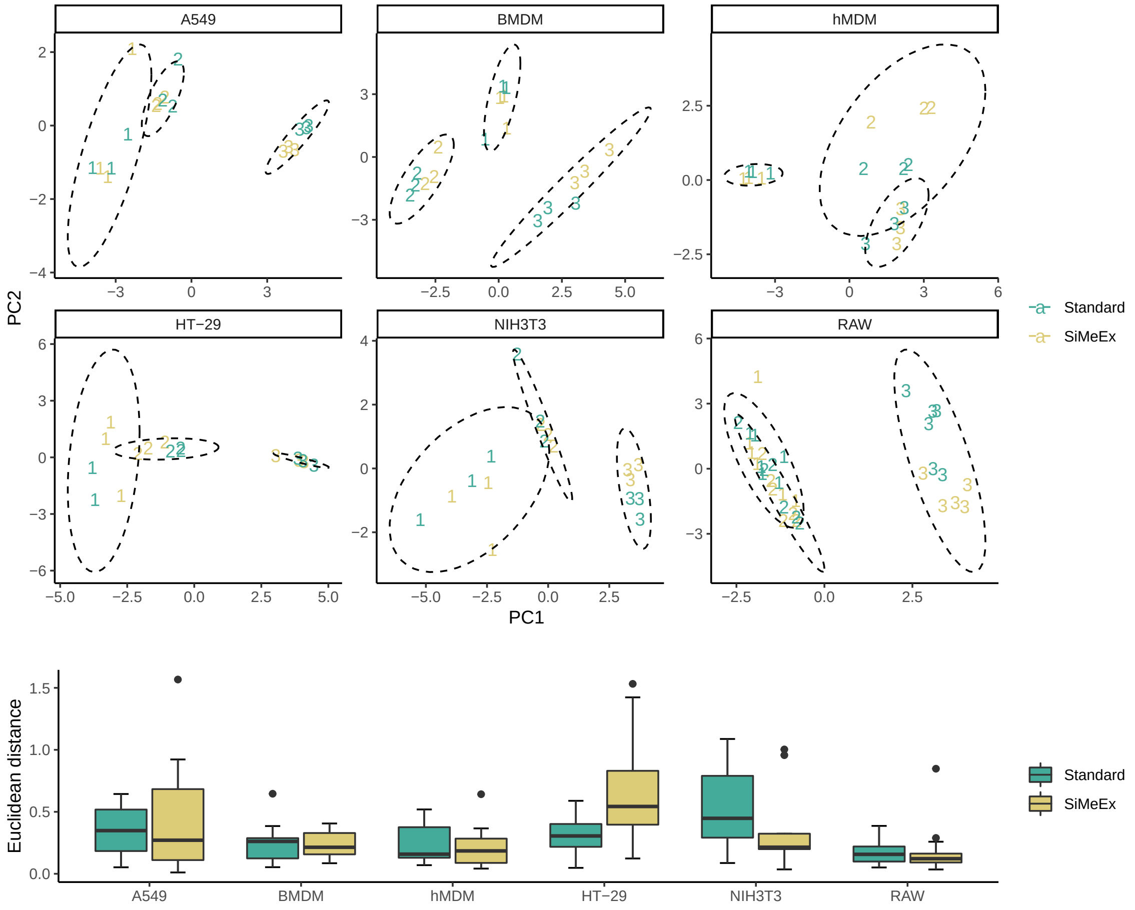

Figure S3. PCA for different cells, targeted analysis. PCA analysis of the extraction methods. Data of a targeted measurement with 12 metabolites as features is used, normalized by internal standard and sum of metabolite signals. Upper plots show scatter plots for cell lines. The colors depict the extraction method and the numbers indicate the biological replicates (6 for RAW264.7 cells and 3 for all other cell lines and primary cells. Ellipse are shown with dashed lines, where the biological replicate is clustered. The reproducibility of both methods is quantified, using the euclidean distance to the group centroid for each biological experiment.
